# Treatment of recurrent pregnancy loss by Levothyroxine in women with high Anti-TPO antibody 

**Published:** 2012-07

**Authors:** Mohammad Hosein Mosaddegh, Nasrin Ghasemi, Tahere Jahaninejad, Fatemeh Mohsenifar, Abbas Aflatoonian

**Affiliations:** 1 *Department of Pharmacology, Shahid Sadoughi University of Medical Sciences, Yazd, Iran.*; 2 *Department of Medical Genetics, Research and Clinical Centre for Infertility, Shahid Sadoughi University of Medical Sciences, Yazd, Iran.*; 3 *Shahid Sadoughi University of Medical Sciences, Yazd, Iran.*; 4 *Yazd Azad University, Yazd, Iran.*; 5 *Department of Obstetrics and Gynecology, Research and Clinical Centre for Infertility, Shahid Sadoughi University of Medical Sciences, Yazd, Iran.*

**Keywords:** *Recurrent pregnancy loss*, *Levothyroxine*, *Anti-TPO antibody*

## Abstract

**Background: **Recurrent pregnancy loss (RPL) is defined as two or more consecutive pregnancy losses before twenty weeks of gestation. It is caused by a variety of genetics and non-genetics factors. Thyroid autoimmunity could associate with pregnancy loss.

**Objective:** To investigate the effectiveness of Levothyroxine in treatment of RPL in women with high auto-thyroid antibodies.

**Materials and Methods:** In this observational cross sectional study, 900 women who had a history of recurrent pregnancy loss were studied. All women with high anti-TPO antibody without any other problems entered in this study. Levothyroxine was given to them two months before pregnancy till the end of pregnancy. The doses of levothyroxine were depended on the anti-TPO levels, which were decided by endocrinologist. Women followed for the results of pregnancies.

**Results:** The success rate of pregnancy in women with abnormal anti-TPO with Levothyroxine therapy was 82.85%. Mean of anti-TPO in women with treatment before taking medication was 488.35 and after that it was 123.35 UI/ml. This difference was significant (p<0.05). The mean of the antibodies was not significantly different in women without treatment.

**Conclusion:** This study showed that Levothyroxine reduces the incidence of spontaneous abortions in women with high Anti-TPO antibody. It decreased anti-TPO antibody levels after 2-3 months treatment.

## Introduction

Recurrent pregnancy loss (RPL) is defined as two or more consecutive pregnancy losses before twenty week of gestation, which affects 1-3% of couples. It is caused by variety of genetics and non-genetics factors. Genetic disorders, reproductive tract anatomical pathologies, infectious diseases, endocrine dysfunctions, thrombophilia and autoimmune diseases are known to be the most important risk factors for RPL (1-6).

There is evidence that thyroid autoimmunity is associated with pregnancy loss, because maternal thyroid hormones (TH) play a critical role in the development of both fetus and placenta (7). Pregnancy loss in women with positive thyroid autoantibodies occurs within the first trimester of gestation, when the fetus is dependent on maternal thyroid hormones (8, 9). Following implantation, the preservation of the pregnancy is reliant on a mass of immunological events that will assistance in the successful growth and development of the fetus (7). 

So, Thyroid autoantibodies generally enhanced autoimmune reactivity against the feto-placental unit as a consequence of hypothyroidism (10). The presence of thyroid autoantibodies can cause infertility and delay pregnancy. Women with high concentration thyroid autoantibodies in their blood circulation usually become pregnant in older age and encounter with a higher risk of pregnancy loss (11-13). 

In the other hand, the presence of thyroid autoantibodies in women during pregnancy could be associated with a lack in thyroid hormone concentrations or a poorer ability of the thyroid gland to sufficiently conform to the requests of pregnancy. Treatment of thyroid insufficiency, caused by auto antibodies, during pregnancy is important in avoiding hostile maternal and fetal outcome (14). 

This study assessed the effect of Levothyroxine therapy on the live-birth rate in women with a history of at least two recurrent unexplained pregnancy loss, which were anti-TPO antibody positive. It also compared anti-thyroid peroxidase antibodies levels before and after treatment.

## Materials and methods

This project evaluated 900 women with recurrent pregnancy loss. They had referred to the Recurrent Abortion Clinic of Yazd Reproductive Sciences Institute. They were evaluated for uterine and cervical anatomical disorders (using pelvic ultrasonography or hysteroscopy), ovarian dysfunction, chromosome abnormality (using conventional karyotype), and then evaluated for thyroid disorders, and autoimmune antibodies. This study approved by ethic committee of yazd Reproductive Science Institute. 

Women with positive thyroid peroxidase antibodies (anti-TPO) and normal TSH were entered to this study. Thyroid peroxidase (TPO) was tested with a chemiluminescence immunoassay, and women with anti-TPO more than 40 UI/ml were treated with levothyroxine after signing inform consent.

After two months anti-TPO was tested again. Levothyroxine doses were depended on the levels of anti-TPO, which were decided by endocrinologist. It was 25-100 μg every day. Treatment continued with levothyroxine and aspirin till pregnancy happened and these continued during pregnancy until delivery. All women were followed during pregnancy to the end. 

**Statistical analysis**

Mean of anti-TPO antibodies in each group were tested before and after 2 months with paired t-test using SPSS (version 16) and p-value<0.05 is significant.

## Results

Forty five unexplained RPL women had anti-TPO more than 40 UI/ml with normal TSH. Mean of anti-TPO in women without treatment and with treatment before pregnancy were 462.45 and 488.35 respectively, which was not significantly different (p0.05). Mean of anti-TPO in women after treatment was 123.35. The difference between mean before and after treatment was significant (p<0.05) (Table I). 

Thirty nine women received Levothyroxine and six women never used Levothyroxine by their own decision. Four of 39 cases never get pregnant (in follow up time). From the remaining 35 cases treated with Levothyroxine, 14 had normal delivery, 9 passed 20 weeks of gestation, 6 had preterm labors with normal child, and 6 had miscarriages. Three of 6 women never used levothyroxine, get pregnant, but all had abortion again. Three of six cases never get pregnant again (Table II).

**Table I:** Mean of anti-TPO in women with recurrent abortion

**Patients**		**Anti-TPO (UI/ml)**	**p-value**
		**Mean± SD**	
With treatment			<0.05
	Before medication	488.35±113.58	
	After 2 months	123.35±38.7	
Without treatment			0.05
	Before medication	462.45±149.33	
	After 2 months	453.12±139.2	

Anti-TPO significantly decreased after treatment with levothyroxine (p-value<0.05).

**Table II:** Success rate of pregnancy in women with abnormal anti-TPO with and without treatment

**Levothyroxine**	**Normal delivery (n)**	**Preterm labor with normal child (n)**	**Pregnancy more than 20 weeks (n)**	**Abortion (n)**	**Sum**	**Successes rate**
Take	14	6	9	6	35	82.85%
Not take	-	-	-	3	3	0%

Pregnancy rate after treatment with levothyroxine in women with high anti-TPO was 82.85%.

## Discussion

Recurrent pregnancy loss is an important clinical problem and classically defined as two or more pregnancy losses before the fetus has reached viability. The leading etiologies associated with it include a variety of causes such as autoimmune diseases (15).

Nilsson *et al* in 1975 found for first time that levels of circulating anticoagulant were higher in women with recurrent pregnancy loss than in control subjects (16).This anticoagulant was later found to be the lupus anticoagulant antibody. Since that time several studies have established the relationship between autoimmune diseases such as anti-phospholipid syndrome, rheumatoid arthritis and systemic lupus erythematous with abortion (17, 18).

A number of studies have revealed relation between thyroid autoantibodies and recurrent abortions. It is suggested that the presence of thyroid autoantibodies could cause a generalized activation of the immune system, which unregulated activity of the immune system at the fetal-maternal interface (19). Lejune *et al* found that frequency of circulating anti-thyroid antibodies were higher in women with recurrent abortion than in control subjects (20). 

In 1990, Stagnaro-Green *et al* tested 552 women for thyroid auto-antibodies in the first trimester of pregnancy. Pregnancy loss rate in women with high antibody was twice than women with normal antibody (10). Regarding prevention of abortion, there are a few studies showing that thyroxin treatment may be efficient in decreasing the number of abortion when given during the early stages of pregnancy. Negro *et al* treated euthyroid women with high thyroid autoantibodies who underwent IVF. 

Half of them received levothyroxine and other half received placebo. The pregnancy loss rate in the placebo group was 52% compared to 33% in the group in which levothyroxine was given. However, because of the small number of investigated patients in their study the difference was not statistically significant (21). In Roussev study, 187 patients were randomized for evaluating the effect of levothyroxine treatment on pregnancy outcomes (22). 

Also in Poppe study levothyroxine was prescribed for 187 women with unexplained recurrent pregnancy loss, and compare live birth rates among two groups (23). In both studies women with normal thyroid function with thyroid autoantibodies were selected. Both studies showed a decrease in pregnancy loss rates (36% and 75% relative reductions). Wang *et al* screened a total of 756 women in the first trimester of pregnancy for thyroid functions after Levothyroxine treatment and then follow up them until delivery. They found that therapy decreased the incidence of spontaneous abortions (24).

## Conclusion

In conclusion, high anti-TPO antibody in women with RPL could increase risk of pregnancy loss, and treatment with levothyroxine helps them to have normal pregnancy.
